# The hepatic protective effect of the association of microencapsulated quercetin and *Bifidobacterium animalis* in colorectal carcinogenesis

**DOI:** 10.1590/1414-431X2026e15588

**Published:** 2026-11-09

**Authors:** C.C.O. Bernardo, L. Casagrande, M.F.T. Carvalho, S.S. Sestak, M.L.M. Sergio, A.L.R.G. Sevilha, S.A. da Silva, L.E.F. Berton, F.C. Flores, Y.M.G. Costa, A.C. Chagas, R. Curi, J.F. Comar, J.N. Zanoni, J.V.C.M. Perles, R.B. Bazotte

**Affiliations:** 1 Programa de Pós-Graduação em Ciências Farmacêuticas, Universidade Estadual de Maringá, Maringá, PR, Brasil; 2 Departamento de Ciências Fisiológicas, Universidade Estadual de Londrina, Londrina, PR, Brasil; 3 Departamento de Ciências Morfológicas, Universidade Estadual de Maringá, Maringá, PR, Brasil; 4 Departamento de Informática, Universidade Estadual de Maringá, Maringá, PR, Brasil; 5 Programa de Pós-Graduação em Bioquímica, Universidade Estadual de Maringá, Maringá, PR, Brasil; 6 Programa de Pós-Graduação Interdisciplinar em Ciências da Saúde, Universidade Cruzeiro do Sul, São Paulo, SP, Brasil; 7 Escola Superior do Instituto Butantan, São Paulo, SP, Brasil; 8 Programa Interdisciplinar em Saúde, Universidade de Marília, Marília, SP, Brasil; 9 Departamento de Bioquímica, Universidade Estadual Maringá, Maringá, PR, Brasil

**Keywords:** Liver, Oxidative stress, Probiotics, Lobar heterogeneity, Deep learning

## Abstract

We investigated the potential of the association between microencapsulated quercetin (MEQ) and the probiotic *Bifidobacterium animalis* in 1,2-dimethylhydrazine (DMH)-induced colorectal cancer (CRC) in Wistar rats with a focus on hepatic alterations and liver lobar heterogeneity. The rats were divided into 5 groups: control (C), CRC (CR), CRC treated with MEQ (CRQ), CRC treated with the probiotic (CRP), and CRC treated with MEQ + probiotic (CRQP). DMH (40 mg/kg) was administered intraperitoneally twice weekly for two weeks. MEQ (10 mg/kg) and the probiotic (5×10^7^ colony forming units) were administered by gavage for 13 weeks. The liver was evaluated by histomorphometry and redox state analyses. CRC was confirmed by aberrant crypt foci in 100% of the DMH animals. The CR group showed increased hepatosomatic index (HSI) and lobe-specific cellular hypertrophy. The treatments reduced the HSI, with the association MEQ + probiotic being the most effective in preventing the physiopathological process associated with CRC. Deep learning-assisted cell quantification revealed a reduction of mononucleated hepatocytes in the CR group, a condition abolished by all treatments. DMH induced asymmetric glycogen accumulation and extracellular matrix remodeling, which were modulated by MEQ and the probiotic. Redox state analysis showed thiol depletion in the caudate lobe, where the quadrate lobe underwent lipid peroxidation and catalase inhibition. The treatments reestablished thiol levels and catalase activity in both lobes, thereby confirming antioxidant and hepatoprotective properties. In conclusion, the association of MEQ and probiotic exhibited lobar heterogeneity in attenuating hepatic damage reinforcing the importance of liver lobar analysis in the therapeutic evaluation of carcinogenesis.

## Introduction

Colorectal cancer (CRC) remains a major global public health challenge, ranking among the neoplasms with the highest incidence and mortality (1). The disease exhibits slow and multifactorial progression, typically initiated by pre-neoplastic lesions (2). In this context, chemopreventive strategies based on the consumption of functional foods and bioactive components have received increasing attention, especially for their potential to modulate cellular pathways involved in tumor initiation and promotion (2,3).

The experimental induction of CRC in animal models, often achieved by administering 1,2-dimethylhydrazine (DMH), reproducibly mimics human pathogenesis and constitutes a validated model for studies of chemopreventive agents. DMH acts as a pro-carcinogen, requiring hepatic metabolism to form highly reactive intermediates (2-4). This metabolism triggers intense oxidative stress, resulting from an imbalance between reactive oxygen species and endogenous antioxidant systems such as reduced glutathione (GSH), catalase (CAT), and superoxide dismutase (SOD) (5-7). Thus, although the primary target of DMH is the colon, the liver is the first organ exposed to DMH and its metabolites and undergoes significant oxidative and structural changes during carcinogenesis (8), making it a relevant target for damage assessment and therapeutic interventions.

Importantly, the colon and liver are anatomically and functionally connected through the gut-liver axis, whereby blood from the intestine drains into the portal vein and reaches the liver carrying nutrients, microbial products, inflammatory mediators, and xenobiotic metabolites. In this setting, changes occurring in the intestinal environment including epithelial injury, dysbiosis, barrier disruption, and local inflammation can secondarily modulate hepatic oxidative stress, inflammatory signaling, and matrix remodeling, and vice versa. Therefore, even when liver damage is initially triggered by DMH metabolism, interventions acting primarily in the intestinal lumen or mucosa may indirectly modify the hepatic response by altering the profile of signals that reach the liver via the portal circulation.

Recently, using the DMH-induced CRC model, our group demonstrated that microencapsulated quercetin and *Bifidobacterium animalis* preserved jejunal enteric neurons and modulated gastrointestinal function during colorectal carcinogenesis (3). Although these treatments did not significantly reduce the formation of aberrant crypts, they attenuated intestinal neurochemical alterations associated with CRC, suggesting an action on inflammatory and oxidative pathways. Building on these findings, the present study extends the investigation to the gut-liver axis, evaluating whether these intestine-targeted interventions can also modulate liver histology, remodel the extracellular matrix, and mitigate disturbances in redox homeostasis in the liver of DMH-exposed animals.

Quercetin, a flavonoid widely distributed in the diet, possesses notable pharmacological properties, including antioxidant, anti-inflammatory, and cytostatic activities (9-11). Its action involves the direct neutralization of free radicals (12) and the modulation of critical signaling pathways in carcinogenesis (13). However, its low oral bioavailability limits its therapeutic efficacy (14). Microencapsulation with biopolymers, such as pectin and casein, emerges as a relevant technological strategy because it protects quercetin from early degradation, improves its solubility and stability, and promotes controlled release in the distal intestine and colon (15,16). In DMH-based models, such targeted delivery is particularly relevant: local availability of quercetin in the colon can attenuate mucosal oxidative stress and inflammation, modulate epithelial and immune cell responses, and potentially influence the composition and activity of the gut microbiota. These local effects may, in turn, alter the quality and quantity of pro-oxidant and pro-inflammatory mediators entering the portal circulation, thereby indirectly impacting liver histology and redox balance, even when the original insult to the liver is the systemic metabolism of DMH.

Concurrently, probiotics modulate gut microbiota composition and confer systemic beneficial effects. Anaerobic, gram-positive bacteria of the genus *Bifidobacterium* play a central role in maintaining intestinal homeostasis and have chemopreventive potential against CRC by modulating the microbiota, reducing oxidative stress, and regulating apoptosis (17,18). By strengthening the intestinal barrier and reducing luminal and mucosal inflammation, probiotics can decrease the translocation of endotoxins and other microbial products into the portal vein, thereby lowering hepatic oxidative and inflammatory burden. Moreover, microbial metabolites derived from probiotic activity, such as short-chain fatty acids, may directly modulate hepatocellular metabolism and antioxidant defenses along the gut-liver axis. The synergistic interaction between bioactive compounds and probiotics has been reported, as observed in the association between quercetin and *Bifidobacterium adolescentis*, which demonstrated an amplification of anti-inflammatory activity in an *in vitro* study (19). Together, these data support the concept that interventions acting primarily in the intestine can exert measurable effects on liver histology and redox state, even in experimental conditions where all DMH-exposed animals develop CRC.

Based on this evidence, we hypothesized that the combination of microencapsulated quercetin (MEQ) and *Bifidobacterium animalis* would enhance CRC chemoprevention and, simultaneously, attenuate liver damage by coupling improved colonic delivery of the flavonoid with the microbiota-modulating effects of the probiotic along the gut-liver axis. In the present study, all DMH-treated groups (regardless of receiving MEQ and/or the probiotic) were submitted to the same carcinogenic protocol and therefore shared exposure to DMH metabolism. Within this context, we specifically asked whether MEQ and *Bifidobacterium animalis* acting predominantly in the intestine could modify the pattern of hepatic injury induced downstream of DMH. Thus, we compared the efficacy of isolated and combined treatments in Wistar rats subjected to DMH-induced carcinogenesis, with emphasis on liver protection. The analyses encompassed biometric parameters, histomorphometry, extracellular matrix remodeling, as well as a segmented assessment of redox status in the liver lobes, integrating these findings with classical colonic alterations to clarify how intestinally acting interventions can reshape liver histology and oxidative responses in CRC.

## Material and Methods

### Animals and experimental conditions

The experimental protocol was conducted in accordance with current ethical standards and approved by the Ethics Committee on the Use of Animals in Experimentation (CEUA) of Maringá State University (Certificate No. 1126010419). Forty male Wistar rats (*Rattus norvegicus*), 43 days old, were used and maintained in polypropylene cages under controlled conditions (22±2°C; 12 h light/dark cycle) with *ad libitum* access to water and food. Animals underwent a seven-day acclimation period during which they received prophylactic antiparasitic treatment via gavage: a single dose of fenbendazole (50 mg/kg, Panacur^®^, MSD Saúde Animal, Brazil) and metronidazole (500 mg/kg per day; Flagyl^®^ Pediátrico, Sanofi Medley Farmacêutica, LTDA, Brazil) for five days, followed by one rest day.

The experimental period began at 50 days of age and lasted 98 days. Animals were randomly distributed into five groups: control (C); CRC (CR); CRC treated with MEQ (CRQ); CRC treated with *Bifidobacterium animalis* probiotic (CRP); and CRC treated with MEQ combined with *Bifidobacterium animalis* probiotic (CRQP). CRC was induced by DMH intraperitoneally (*ip*) administered (40 mg/kg), twice weekly for two weeks (20).

### Treatment preparation

MEQ was produced from pectin/casein dispersion (11,16,21) and characterized by transmission electron microscopy (TEM) and X-ray diffraction. The particles exhibited irregular morphology (20-500 µm), distinct from the acicular crystalline pattern of pure quercetin (15). The probiotic *Bifidobacterium animalis* subsp. *Lactis* was acquired from Active Pharmaceutica (Brazil).

### Treatment and sample collection

Following CRC induction, animals received daily gavage treatments using in deionized water. MEQ was administered at a dose of 10 mg/kg bw (11) and the probiotic at 5×10^7^ CFU (colony forming units) (18). Doses were adjusted individually every two days according to body mass.

At the end of the experimental period, animals were weighed and anesthetized with lidocaine (10 mg/kg, *ip*) and thiopental (150 mg/kg, *ip*), then euthanized by anesthetic overdose, and celiotomy was performed for liver removal of right lobe (RL), left lobe (LL), caudate lobe (CL), and quadrate lobe (QL).

### Confirmation of the experimental model

CRC induction was confirmed by quantification of aberrant crypt foci (ACF). This experimental model and the methodology for ACF analysis have been previously established and validated in our laboratory (3). Segments of the proximal and distal colon were opened longitudinally along the mesenteric border, fixed in 4% paraformaldehyde, transferred to 70% ethanol, and stained with 1% methylene blue (Sigma-Aldrich, USA) in 0.1 M phosphate-buffered saline (PBS), pH 7.4. ACF were evaluated along the entire length of each colonic segment under light microscopy at 40× magnification. Aberrant crypts were identified according to the morphological criteria originally described by Bird (22), including enlarged and slightly elevated epithelial borders, oval- or slit-shaped lumens, and increased staining intensity compared with adjacent normal colonic crypts. In the present study, results are reported as ACF, defined as groups containing two or more aberrant crypts. Analyses were performed across the full length of the proximal and distal colon segments without normalization by surface area. To minimize potential bias associated with differences in colonic dimensions, colon length was measured during sample collection, and no significant differences in total colon length were observed among the experimental groups.

### Determination of biometric parameters

Body mass was measured at baseline, every two days during the experiment, and two hours before euthanasia. The liver was removed, weighed, and immediately sectioned for specific analyses. Body mass variation (ΔBM) was calculated as the difference between the final and initial mass. The hepatosomatic index (HSI) was calculated to express the proportion of organ mass relative to the animal's final body mass using the formula: HSI = (liver mass/body mass) × 100.

### Sample preparation for histological analysis and image acquisition

The lobes were rinsed in PBS (0.1 M; pH 7.4), fixed in 10% buffered formalin for 18 h, dehydrated in a graded ethanol series, cleared in xylene, and embedded in paraffin. Semi-serial sections of 5-μm thickness were obtained using a microtome (Leica RM2125 RTS, Germany) and stained with hematoxylin-eosin (HE), periodic acid-Schiff (PAS), and Picrosirius red.

HE and PAS images were captured using a Moticam^®^ 2500 (Motic, China) camera coupled to a Motic BA400 (Motic) microscope. Picrosirius-red images were obtained using a Roper Scientific Photometrics^®^ camera (Roper Scientific/Photometrics, USA) coupled to an Olympus BX-53 (Japan) microscope with polarizer. All images were transferred to a computer using Motic ImagesPlus^®^ 2.0 and analyzed using Image-Pro Plus^®^ 4.5 (Media Cybernetics Inc., USA).

### Morphometric analysis of hepatocytes

Ten images per animal (40× objective) were captured, distributed across two fields per section and five sections per animal. Images were randomly obtained from the midzonal hepatic region, located between the periportal and perivenous areas of the hepatic lobule. One hundred hepatocytes with well-defined boundaries were randomly selected for measurement of cell and nuclear areas (μm^2^) using Image-Pro Plus^®^ 4.5. The cytoplasmic area was obtained by the difference between these measurements, and the nucleus-to-cytoplasm ratio was calculated (23).

### Quantitative analysis of hepatocytes

Hepatocyte quantification was performed using the same set of images obtained for morphometric analysis, ensuring sample consistency. Cell counting was conducted using a deep learning-based artificial intelligence tool that integrates the You Only Look Once (YOLO) algorithm for nuclear detection and the Segment Anything Model (SAM) for precise segmentation of structures (23). Images were automatically processed, generating marking maps in which mononucleated hepatocytes are identified in green and binucleated hepatocytes in yellow. This comprehensive processing of each field ensures greater accuracy in determining the proportion of mono- and binucleated hepatocytes. The tool generates, at the end, a structured file containing detailed quantification by image and experimental group.

### Quantification of hepatic glycogen

PAS-stained sections were analyzed across five sections per animal, selecting six fields per section under a 40× objective, totaling 30 images per animal and 240 images per group. Glycogen content was quantified by colorimetric analysis of PAS-positive areas using Image-Pro Plus^®^ software and expressed as the percentage of occupied area, according to Azevedo et al. (24).

### Quantification of type I and III collagen in liver tissue

Collagen type quantification was performed on sections stained with Picrosirius red under polarized light. Picrosirius red is a selective connective tissue stain that enables qualitative assessment of collagen fibers within connective tissue. When examined under polarized light, differences in color interference, intensity, and tissue birefringence allow this stain to effectively differentiate between type I and type III collagen fibers. For each animal, five sections were evaluated, and from each section, 16 fields were photomicrographed at 20× objective, following a systematic path with exclusion of intermediate fields (25).

### Assessment of oxidative status

For analyses of thiobarbituric acid-reactive substances (TBARS), total thiols, and catalase (CAT), the lobes of frozen liver were homogenized in a Potter-Elvehjem homogenizer with ten volumes of ice-cold 0.1 M potassium phosphate buffer (pH 7.4). An aliquot of the material was separated as total homogenate, while the remainder was centrifuged (11,000 *g*, 15 min, 4°C) to obtain the soluble fraction. For determination of lipid hydroperoxide (LOOH) levels, the same hepatic lobes were homogenized in 200 mM potassium phosphate buffer (pH 6.5) in a 1:10 (w/v) ratio, followed by centrifugation at 11,000 *g* for 20 min at 4°C; the resulting supernatant was collected and aliquoted (200 µL) for LOOH analysis.

Total protein content was determined in the homogenate supernatant using the Folin phenol reagent (26).

LOOH levels were quantified, and absorbance was measured at 560 nm; LOOH concentrations were calculated using an extinction coefficient of 4.3 mM/cm. Results are reported as mmol/mg tissue (27).

TBARS levels were quantified by spectrophotometry (502 nm) (28). Standard curves were prepared with 1,1',3,3'-tetraethoxypropane, and values are reported as nmol/(g tissue).

The content of total sulfhydryl groups (thiols) in the homogenate supernatant was determined by spectrophotometry (412 nm) using 5,5'-dithiobis (2-nitrobenzoic acid) (DTNB) (29). Data are reported as µmol/g tissue.

Catalase activity was estimated by the change in absorbance at 240 nm using hydrogen peroxide (H_2_O_2_) as substrate, and results are reported as mmol/(min × mg protein) (30).

### Statistical analysis

Statistical analysis was conducted using GraphPad Prism^®^ 8.0 (USA) and Statistica^®^ 10.0 software (USA). Normality of the distribution was verified using the Shapiro-Wilk test. For comparisons between hepatic lobes, Student's *t-*test was applied when data showed normal distribution, and the Mann-Whitney test was used for non-normal distributions. For comparisons between experimental groups within the same lobe, block design analysis followed by Fisher's *post hoc* test was employed. Data are reported as means±SE, with statistical significance considered when P<0.05.

## Results

### Confirmation of the experimental model

The induction of carcinogenesis was confirmed by the presence of ACF in 100% of the animals exposed to DMH, in both the proximal and distal colon (Figure 1C). Morphological analysis using methylene blue staining revealed distortion of the cryptic lumen and epithelial thickening characteristic of pre-neoplastic lesions (Figure 1A and B).

**Figure 1:**
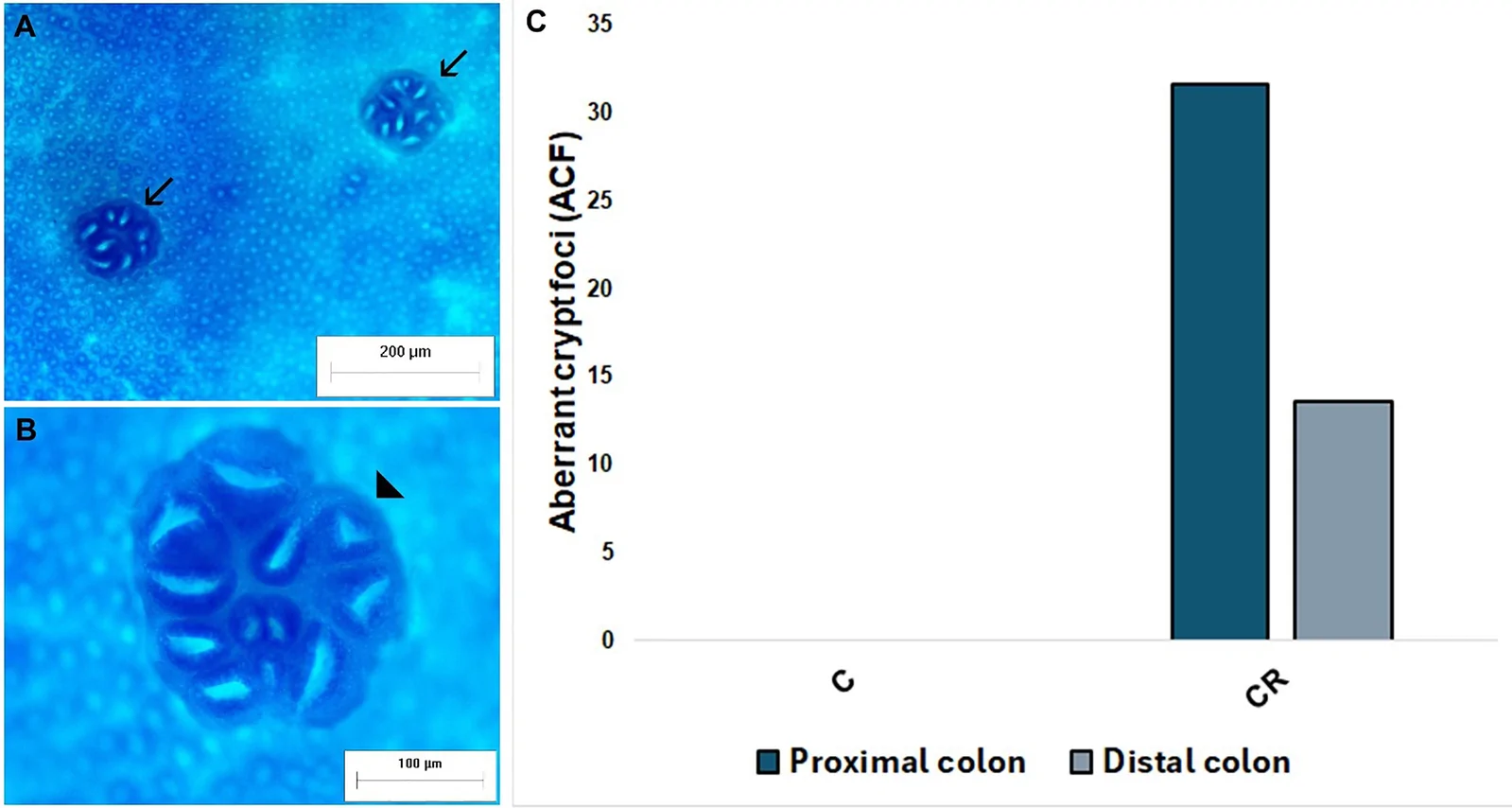
The 1,2-dimethylhydrazine (DMH)-induced carcinogenesis model triggered the formation of aberrant crypt foci (ACF) in the proximal and distal colon of rats. Representative photomicrographs of ACF (arrow and arrowhead) in the colon stained with methylene blue at 10× magnification, scale bar=200 µm (**A**); 40× magnification, scale bar=100 µm (**B**); and quantification graph (**C**). Experimental groups: control (C, n=8) and colorectal carcinogenesis (CR, n=8).

### Biometry

The CR group showed greater body weight change, absolute liver mass, and HSI than the C group (P<0.05). The CRQ, CRP, and CRQP groups showed a reduction in liver mass and HSI (P<0.05) compared to the CR group. The CRQP group exhibited lower (P<0.05) final body mass, body mass variation, absolute liver mass, and HSI compared to the CR group (Table 1).

**Table 1:** Initial body weight (IBW), final body weight (FBW), body weight change (BWC), liver weight (LW), and hepatosomatic index (HSI).

	C	CR	CRQ	CRP	CRQP
IBW (g)	268.2±9.0	264.7±4.5	253.2±6.8	260.3±3.8	250.2±4.8
FBW (g)	451.7±12.6	477.2±11.6	452.7±20.2	471.5±11.6	423.5±19.8**
BWC (g)	183.5±7.5	212.5±9.7*	199.5±19.9	211.2±14.3	173.2±21.2**
LW (g)	15.7±0.8	20.0±0.63*	14.7±0.61**	15.0±0.7**	12.9±0.6**
HSI (%)	3.5±0.1	4.2±0.2*	3.3±0.1**	3.2±0.07**	3.0±0.09**

Data are reported as means±SE (n=8/group). Experimental groups: control (C), colorectal carcinogenesis (CR), CR treated with quercetin (CRQ), CR treated with probiotic (CRP), and CR treated with quercetin and probiotic (CRQP). *P<0.05 *vs* C, **P<0.05 *vs* CR. Fisher's *post hoc* test.

### Hepatic morphometric alterations and lobar heterogeneity

Morphometric analysis revealed distinct patterns among the hepatic lobes (Table 2). In the RL, the CR group showed an increase in nuclear area and the nucleus-to-cytoplasm ratio compared to the control group (P<0.05). In the LL, the CR group exhibited cellular hypertrophy, characterized by an increase in cellular, nuclear, and cytoplasmic areas (P<0.05), accompanied by a reduction in the nucleus-to-cytoplasm ratio (P<0.05).

**Table 2:** Morphometric assessment of cellular area (CA), nuclear area (NA), cytoplasmic area (CyA), and nucleus-to-cytoplasm ratio (N/C) of hepatocytes in the right lobe (RL) and left lobe (LL).

	C		CR		CRQ		CRP		CRQP	
	RL	LL	RL	LL	RL	LL	RL	LL	RL	LL
CA (µm^2^)	179.9±1.15^#^	159.3±0.74	183.5±1.54	187.3±1.46*	245.5±1.86**	258.6±2.10**	160.1±0.85**	171.2±0.9**	212.1±1.68**	200.1±1.5**
NA (µm^2^)	42.8±0.28	40.3±0.19	47.4±0.44*	44.7±0.40*	54.9±0.41**	54.2±0.42**	42.5±0.24**	41.5±0.30**	51.5±0.46**	47.1±0.36**
CyA (µm^2^)	137.1±1.04^#^	118.9±0.71	136.0±1.29	142.7±1.24*	190.6±1.68**	204.4±1.91**	117.6±0.79**	129.7±0.83**	160.6±1.45**	153.0±1.3**
N/C	0.32±0.002	0.35±0.002	0.36±0.003*	0.32±0.003*	0.30±0.002**	0.28±0.003**	0.37±0.003**	0.33±0.003	0.33±0.003**	0.32±0.002

Data are reported as means±SE (n=8/group). Experimental groups: control (C), colorectal carcinogenesis (CR), CR treated with quercetin (CRQ), CR treated with probiotic (CRP), and CR treated with quercetin and probiotic (CRQP). *P<0.05 *vs* C, **P<0.05 *vs* CR; Fisher's *post hoc* test. ^#^P<0.05 C-RL *vs* C-LL; Student's *t*-test.

The treatments modulated these parameters differentially. An increase in cellular area in the RL was observed in the CRQ and CRQP groups compared with CR (P<0.05). In contrast, treatment with the probiotic alone (CRP group) resulted in a reduction in cellular, nuclear, and cytoplasmic dimensions in both evaluated lobes (P<0.05).

### Quantification of mononucleated and binucleated hepatocytes in the RL and LL

The CR group showed a reduction in the quantity of mononucleated hepatocytes in the RL and LL (P<0.05). All treatments (CRQ, CRP, or CRQP) abolished this reduction (P<0.05) (Figure 2A). Additionally, an increase in binucleated hepatocytes in the RL was observed in the CRQ and CRP groups (P<0.05) (Figure 2B).

**Figure 2:**
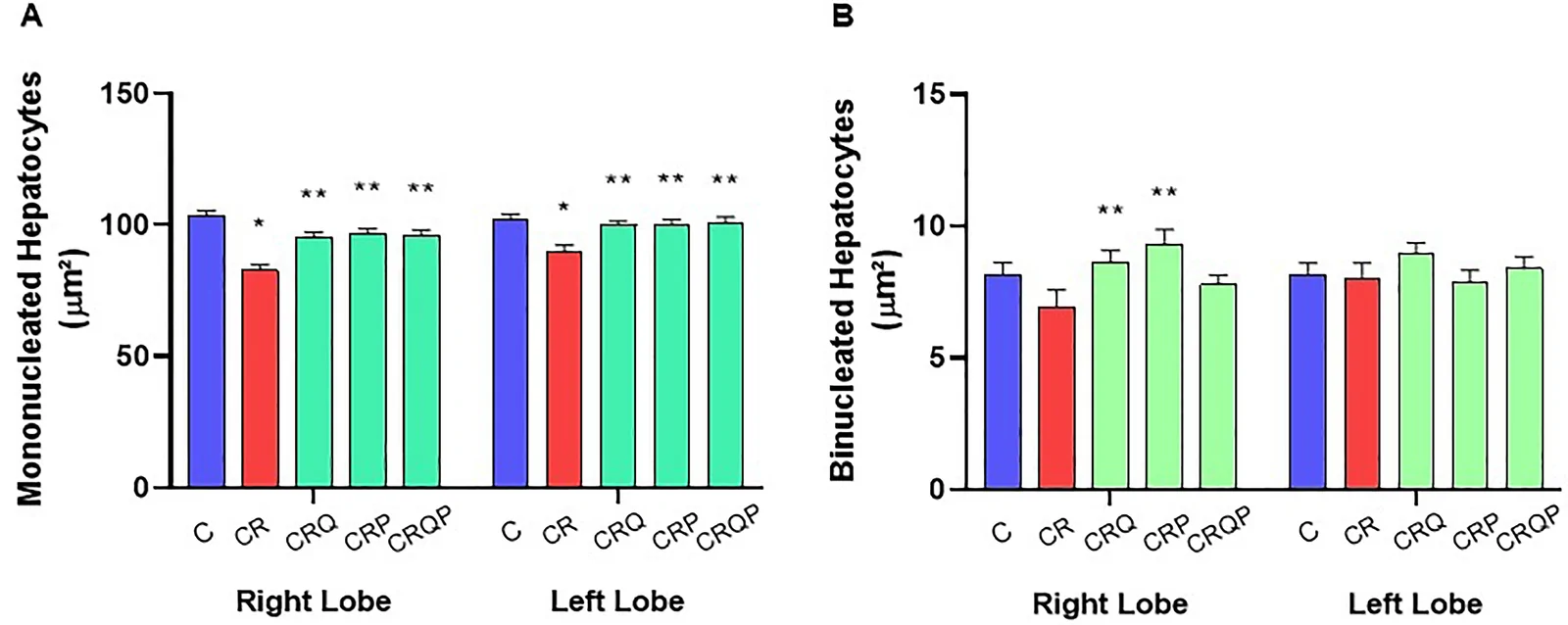
Quantitative assessment of mononucleated (**A**) and binucleated (**B**) hepatocytes in the right and left lobes. Experimental groups: control (C), colorectal carcinogenesis (CR), CR treated with quercetin (CRQ), CR treated with probiotic (CRP), and CR treated with quercetin and probiotic (CRQP). Data are reported as means±SE (n=8/group). *P<0.05 *vs* C, **P<0.05 *vs* CR; Block design analysis followed by Fisher's *post hoc* test.

### Glycogen content

Exposure to DMH induced accumulation of hepatic glycogen, evidenced by an increase in the area occupied by glycogen in the CR groups (n=8) in both lobes (Figure 3A). In the RL, there was a reduction in glycogen in the CRQ and CRP groups (P<0.05), whereas in the LL, the CRQP, CRQ, and CRP groups maintained elevated levels (P<0.05). Lobar asymmetry was observed with greater deposition in the RL compared to the LL in the C and CR groups (P<0.05).

**Figure 3:**
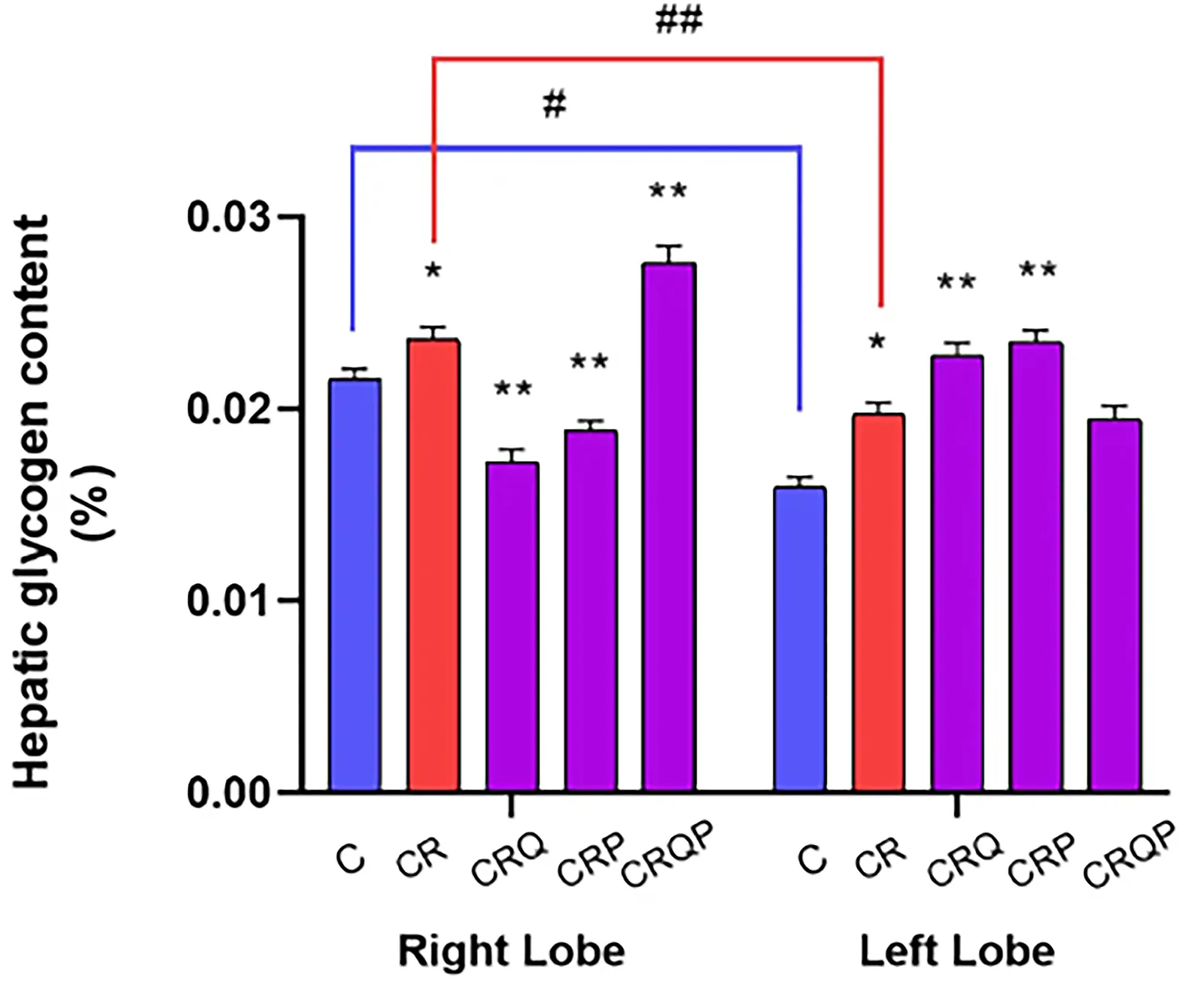
Liver glycogen content in the right and left lobes (RL, LL). Experimental groups: control (C), colorectal carcinogenesis (CR), CR treated with quercetin (CRQ), CR treated with probiotic (CRP), and CR treated with the combination of quercetin and probiotic (CRQP). Data are reported as means±SE (n=8/group). *P<0.05 *vs* C, **P<0.05 *vs* CR; Fisher's *post hoc* test. ^#^P<0.05, ^##^P<0.05; Mann-Whitney test.

### Analysis of the extracellular matrix (ECM)

DMH did not increase type I collagen compared with the control group (Figure 4A). Interestingly, the CRQ and CRP groups showed a reduction in the area occupied by type I collagen in the RL (P<0.05). However, in the LL, all treatments increased type I collagen deposition relative to the CR group (Figure 4A). Nevertheless, there was significant remodeling: the CR group showed a reduction in the area occupied by type III collagen specifically in the RL (P<0.05) (Figure 5A). Lobar asymmetry was also observed, with greater deposition of type I collagen fibers in the RL compared to the LL in the C and CR groups (P<0.05), and of type III collagen fibers in the RL compared to the LL in the C group (P<0.05).

**Figure 4:**
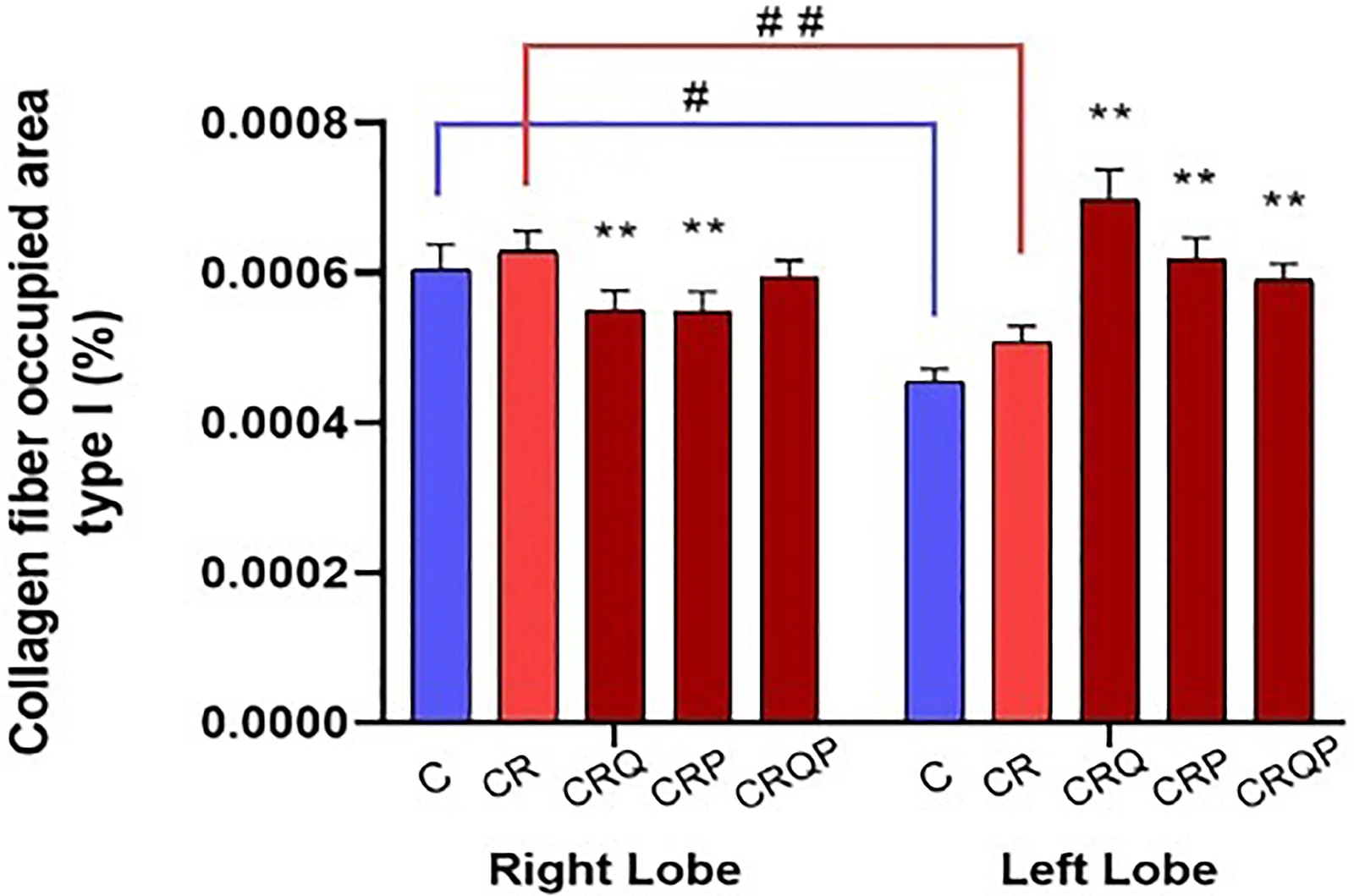
Area occupied by type I collagen in the right and left lobes (RL, LL). Experimental groups: control (C), colorectal carcinogenesis (CR), CR treated with quercetin (CRQ), CR treated with probiotic (CRP), and CR treated with the combination of quercetin and probiotic (CRQP). Data are reported as means±SE (n=8/group). **P<0.05 *vs* CR; Fisher's *post hoc* test. ^#^P<0.05, ^##^P<0.05; Mann-Whitney test.

**Figure 5:**
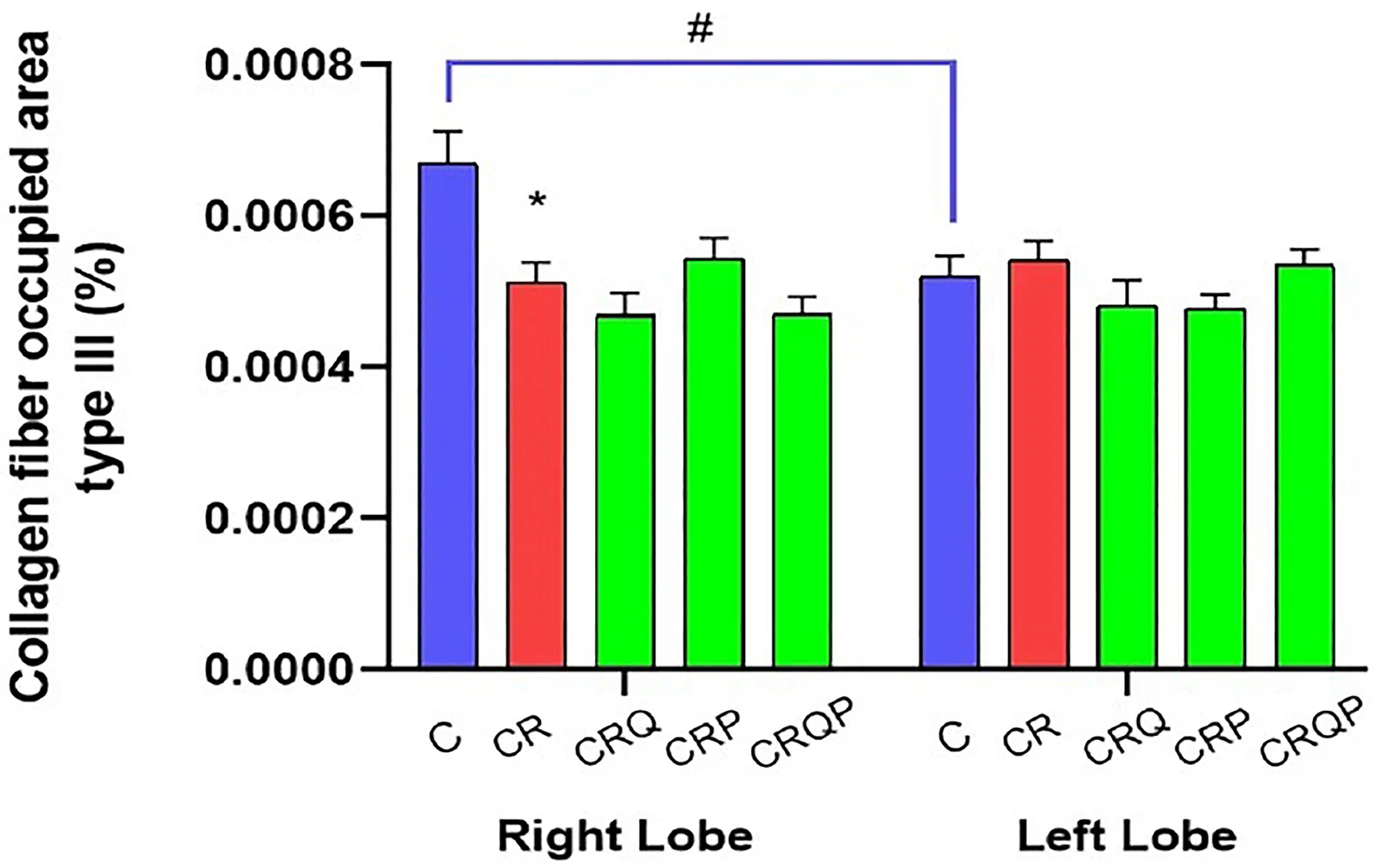
Area occupied by type III collagen in the right and left lobes (RL, LL). Experimental groups: control (C), colorectal carcinogenesis (CR), CR treated with quercetin (CRQ), CR treated with probiotic (CRP), and CR treated with quercetin and probiotic (CRQP). Data are reported as means±SE (n=8/group). *P<0.05 *vs* C; Fisher's *post hoc* test. ^#^P<0.05; Mann-Whitney test.

### Hepatic redox state

The CR group showed an increase in tissue TBARS levels in the QL (P<0.05) (Figure 6A), and a reduction of thiols in the CL (P<0.05) and catalase activity (P<0.05) in the QL (Figure 7A and B). Furthermore, an elevation in TBARS levels was observed in the CL in the CRP and CRQP groups (P<0.05).

**Figure 6:**
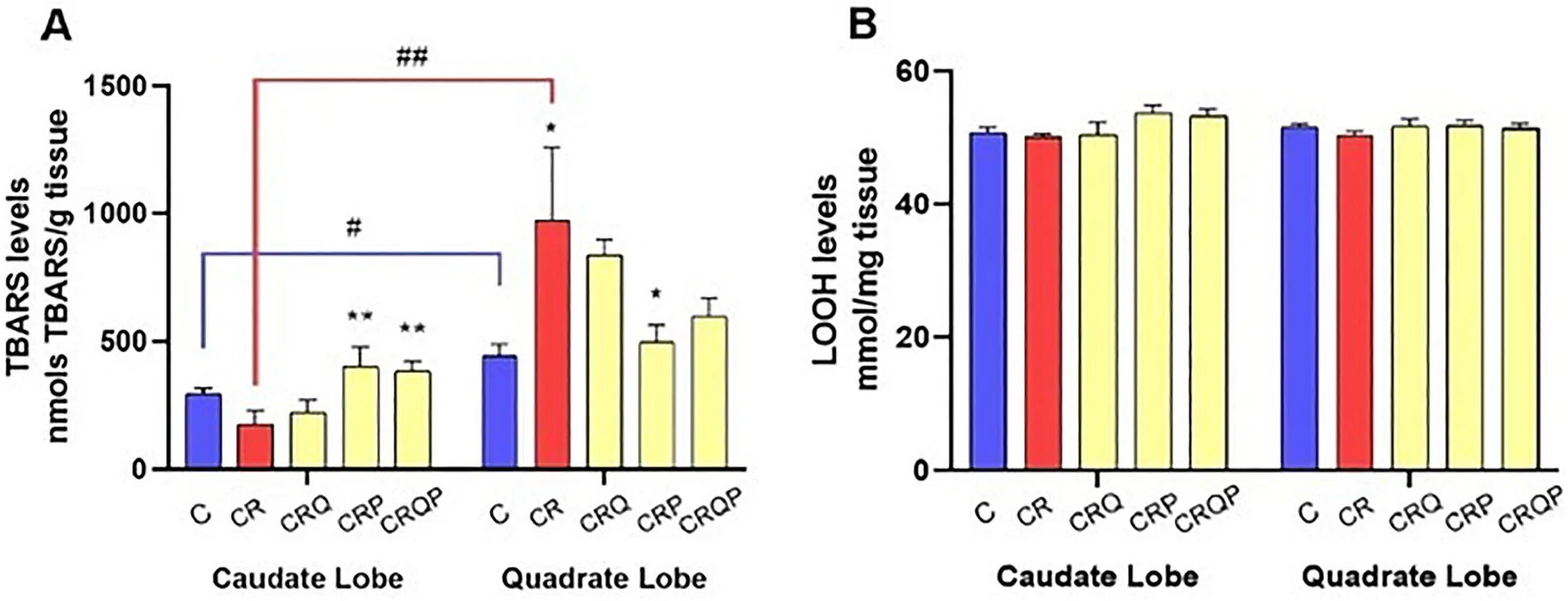
Oxidative damage parameters: thiobarbituric acid reactive substances (TBARS) levels (**A**) and lipid hydroperoxide (LOOH) levels (**B**) in the caudate and quadrate liver lobes (CL, QL). Experimental groups: control (C), colorectal carcinogenesis (CR), CR treated with quercetin (CRQ), CR treated with probiotic (CRP), and CR treated with quercetin and probiotic (CRQP). Data are reported as means±SE (n=8/group). *P<0.05 *vs* C, **P<0.05 *vs* CR; Fisher's *post hoc* test. ^#^P<0.05, ^##^P<0.05; Student's *t*-test.

**Figure 7:**
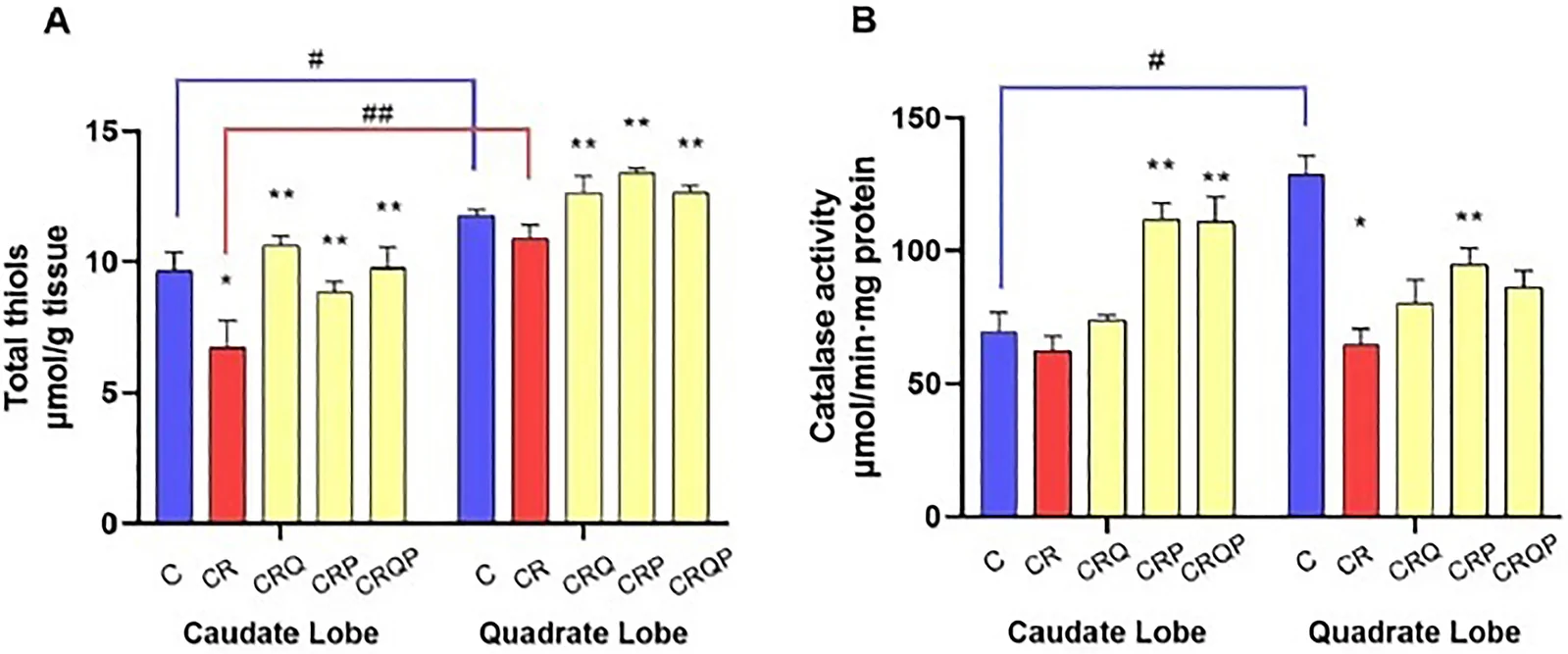
Antioxidant parameters: total thiol group levels (A) and catalase activity (B) in the caudate and quadrate liver lobes (CL, QL). Experimental groups: control (C), colorectal carcinogenesis (CR), CR treated with quercetin (CRQ), CR treated with probiotic (CRP), and CR treated with quercetin and probiotic (CRQP). Data are reported as means±SE (n=8/group). *P<0.05 vs C, **P<0.05 vs CR; Fisher's post hoc test. ^#^P<0.05, ^##^P<0.05; Student's t-test.

There was a reduction in tissue TBARS levels in the CRP group in the QL (P<0.05); recovery of thiol levels in the CRQ, CRP, and CRQP groups in the CL and QL (P<0.05) (Figure 7A); and an increase in catalase activity in the CRP and CRQP groups in the CL (P<0.05). The CRP group reversed the enzyme inhibition induced by the carcinogen in the QL (P<0.05) (Figure 7B).

## Discussion

The present study demonstrated that MEQ and probiotic administered alone or in combination attenuated early hepatic alterations associated with DMH-induced CRC. By integrating histomorphometry, ECM quantification, and segmented redox analyses with deep learning-assisted hepatocyte counting, we showed that both injury and protection were heterogeneously distributed across liver lobes, rather than uniformly expressed. This reinforces the importance of segmented hepatic analyses in carcinogenesis models as demonstrated by our previous studies (31,32).

Model validity was confirmed by ACF in all DMH-exposed animals, in accordance with previous descriptions of this model CRC (4). The CR group exhibited increased HSI, suggesting hepatic metabolic adaptation to carcinogen exposure beyond colonic lesions, as previously reported (3,33). Given that DMH requires hepatic bioactivation and generates reactive intermediates (34), these findings are biologically plausible and align with prior evidence of extra-colonic oxidative/metabolic burden in DMH-based models (7,8). Interestingly, the more pronounced reduction in HSI of the CRQP group suggests a possible additive or synergistic effect of the combined treatment in the liver.

The increased liver mass in CR, without evidence of increased hepatocyte number, is consistent with hepatocellular hypertrophy. Morphometric data support this interpretation, particularly in the LL, where cellular and cytoplasmic expansion was prominent. Such enlargement is consistent with adaptive hepatocellular responses associated with xenobiotic processing and metabolic demand (8). Importantly, the observation that CRP reduced cell, nuclear, and cytoplasmic dimensions in both lobes while CRQ, CRP, and CRQP reduced liver mass and HSI compared with the CR group suggests modulation of DMH-associated hepatocellular adaptive responses.

Deep learning-based quantification added a relevant functional layer to morphometry (24). The reduction in mononucleated hepatocytes in CR (right and left lobes) and its reversal by all treatments suggest preservation of hepatocyte population homeostasis under carcinogenic stress (24). The increase in binucleated hepatocytes in treated groups (notably RL in CRQ and CRP) may indicate adaptive hepatocellular polyploidization rather than worsening injury, especially when interpreted alongside lower HSI and improved antioxidant markers. Although the functional meaning of binucleation in this context requires mechanistic confirmation, this pattern is compatible with adaptive hepatocellular responses.

DMH exposure also induced lobe-asymmetric glycogen accumulation consistent with reduced activity of glucose-6-phosphatase (35). This lobar asymmetry distribution reinforces the idea that different lobes are differentially engaged in metabolic adaptation to carcinogenic stress and treatment.

In the RL, both quercetin and the probiotic reduced glycogen accumulation, which may reflect improved glucose metabolism (36,37). In contrast, in the LL, glycogen content remained elevated in treated groups, particularly for quercetin. This pattern might be related to quercetin-mediated inhibition of GSK-3β and subsequent activation of glycogen synthase (36).

Redox findings provide mechanistic support for structural outcomes. CR animals showed increased TBARS in the QL and thiol depletion in the CL, along with reduced catalase activity in the QL, suggesting compartmentalized oxidative imbalance. This asymmetric profile is coherent with functional hepatic zonation and portal stream distribution, in which lobes may receive distinct mixtures of intestinal and systemic mediators (38). In agreement with this affimation, our recent studies highlight that susceptibility to metabolic and oxidative damage varies across hepatic lobes in models of liver steatosis (31,32). Our data extend this concept to a CRC model and indicate that the CL and QL exhibit distinct redox response to DMH.

Quercetin, owing to its polyphenolic structure, directly scavenges reactive oxygen species and induces antioxidant responses (12). The probiotic acts as a redox modulator, reducing oxidative stress and inflammation through multiple mechanisms, including improved intestinal barrier function and reduced lipopolysaccharide translocation to the liver (39). In our model, probiotic treatment increased catalase activity in both lobes and reversed DMH-induced catalase inhibition in the QL, suggesting partial normalization of site-specific antioxidant defenses. The increase in TBARS in the CL of CRP and CRQP, despite overall recovery of thiols and catalase, suggests spatially and temporally heterogeneous redox adaptation rather than uniform oxidative worsening; this interpretation, however, requires time-course and mechanistic confirmation.

Regarding ECM, DMH did not increase type I collagen globally, indicating that under the present conditions the model did not progress to overt fibrotic remodeling. However, the reduction of type III collagen in RL in CR suggests early matrix disorganization. Type III collagen contributes to tissue architecture, and its degradation has been associated with tissue disorganization and lesion progression (40). Increased type I collagen in LL occurred in treated groups, concomitantly with improved redox status and reduced HSI, favoring adaptive extracellular matrix remodeling rather than overt fibrotic progression. Still, without additional fibrogenic markers, this conclusion should remain cautious.

Taken together, these findings show that MEQ and *Bifidobacterium animalis* act beyond generic antioxidant/anti-inflammatory effects, promoting lobe-specific modulation of hepatic structure and function in CRC. The combined treatment (CRQP) provided the most consistent benefit, reducing HSI and restoring thiol content and catalase activity, in line with our hypothesis of enhanced chemopreventive and beneficial modulation of hepatic oxidative and structural alterations. This effect is biologically plausible: microencapsulation improves colonic delivery of quercetin and its local anti-inflammatory/antioxidant activity (15,16), whereas probiotics modulate microbiota composition and barrier integrity, altering the portal influx of microbial metabolites and endotoxins (17-19,35,39). Their convergence along the gut-liver axis likely underlies the superior response observed with the combined intervention.

From a translational standpoint, the demonstration of clear interlobar heterogeneity in response to DMH and to chemopreventive interventions has important implications. Several studies evaluate the liver as a homogeneous organ, using whole-liver samples or single-lobe biopsies, potentially overlooking regional differences in damage and therapeutic response (36-38). Our results support the notion that segmented analysis of hepatic lobes can reveal protective or deleterious effects that would otherwise be diluted in global measurements. This is particularly relevant for the evaluation of nutritional, pharmacological, and microbiota-targeted strategies intended to modulate the gut-liver axis in cancer and metabolic diseases.

This work has limitations. First, we focused on early carcinogenic alterations and hepatic changes. Second, microbiome composition, bacterial metabolites (e.g., short chain fatty acids, SCFAs), endotoxemia markers, and portal inflammatory mediators were not directly measured; therefore, gut-liver axis mechanisms are biologically plausible but inferential. Third, redox analyses were performed in selected lobes and with limited sample size. Third, only male rats and a single experimental time point were used. Fourth, the functional consequences of changes in binucleation and ECM composition remain to be fully elucidated. Future studies should integrate longitudinal microbiome/metabolomic profiling, inflammatory mediators, fibrosis panels, and functional hepatic readouts to define causality and temporal dynamics more precisely.

In summary, MEQ and probiotic attenuated early DMH-associated hepatic alterations in CRC, in a lobe-dependent manner. The combined treatment provided the most consistent protection across biometric and redox parameters. These results underscore the potential of gut-targeted strategies for secondary liver protection and establish interlobar heterogeneity as a key variable in experimental hepatology and translational chemoprevention.

## Data Availability

All data generated or analyzed during this study are included in this published article
